# Modification of Marine Natural Product Ningalin B and SAR Study Lead to Potent P-Glycoprotein Inhibitors

**DOI:** 10.3390/md12105209

**Published:** 2014-10-17

**Authors:** Chao Yang, Iris L. K. Wong, Wen Bin Jin, Tao Jiang, Larry M. C. Chow, Sheng Biao Wan

**Affiliations:** 1 Key Laboratory of Marine Drugs, Ministry of Education, Shandong Provincial Key Laboratory of Glycoscience & Glycotechnology, School of Medicine and Pharmacy, Ocean University of China, Qingdao 266003, China; E-Mails: yangchao.hy@163.com (C.Y.); wenbinjin0418@gmail.com (W.B.J.); jiangtao@ouc.edu.cn (T.J.); 2 Department of Applied Biology and Chemical Technology, The Hong Kong Polytechnic University, Hung Hom, Hong Kong, China; E-Mail: iris.l.k.wong@polyu.edu.hk

**Keywords:** ningalin B analogues, multidrug resistance (MDR), pyrrole, P-glycoprotein, P-gp modulators

## Abstract

In this study, new marine ningalin B analogues containing a piperazine or a benzoloxy group at ring C have been synthesized and evaluated on their P-gp modulating activity in human breast cancer and leukemia cell lines. Their structure-activity relationship was preliminarily studied. Compounds **19** and **20** are potent P-gp inhibitors. These two synthetic analogues of permethyl ningalin B may be potentially used as effective modulators of P-gp-mediated drug resistance in cancer cells.

## 1. Introduction

Multidrug resistance (MDR) has been a severe problem in clinic cancer chemotherapy. Overexpression of P-glycoprotein (P-gp) drug efflux pump is one of the major mechanisms of MDR. P-gp pumps a wide variety of anti-cancer drugs out of the cells and hereby results in less intracellular drug accumulation [1,2,3]. Three generations of P-gp inhibitors have been developed with a goal of reversing cancer MDR [3,4]; however, only a few non-toxic and P-gp specific inhibitors have been found and none of these inhibitors can be used clinically [5].

**Figure 1:**
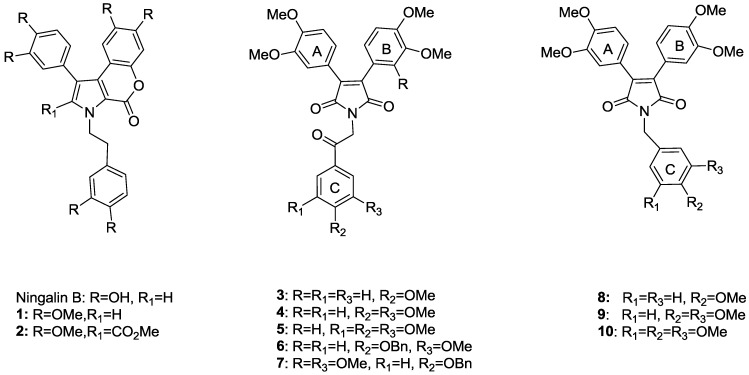
Known analogues of ningalin B acting as P-gp inhibitors.

A class of pyrrole-containing marine natural products such as lamellarins [6,7,8], ningalins [9,10,11], baculiferins [12], polycitrins [13], polycitones [13,14], storniamides [15], didemnimides [16], and purpurones [17] have been isolated in recent years. They were found to show various biological activities including antioxidant [18], antitumor [19,20,21], antivirus [12], and antibacterial [22]. Importantly, some of these pyrrole-containing marine natural products exhibit potential multidrug resistance reversal activity [3,23,24]. Ningalin B, (Figure 1) which is the second member of ningalin family, was isolated and characterized by Fenical’s group from an ascidian of the genus *Didemnum* collected in western Australia near Ningaloo Reef in 1997 [11]. Total synthesis of ningalin B was accomplished in 2000 [9]. It has been found that synthetic permethyl ningalin B (**1**) and its derivatives **2** (Figure 1) which possess a 3,4-diaryl-substituted pyrrole nucleus bearing a 2-carboxylate, are more effective than verapamil in reversing MDR by inhibiting the functionality of P-gp [23].

In our previous study, we have replaced the scaffold of permethyl ningalin B with pyrrole-2,5-dione and obtained a group of 3,4-diarylpyrrole-2,5-diones (such as compounds **3**–**7** of series A and compounds **8**–**10** of series B shown in Figure 1) [23,24]. The modified permethyl ningalin B analogues are more stable and easier to synthesize than permethyl ningalin B [25]. Their MDR reversal activity has been improved [23]. After structure-activity relationship study, two lead compounds **6** and **7** (shown in Figure 1) with a benzoloxy group at ring C and a carbonylmethylene linker at N were demonstrated to be potent P-gp inhibitors [23]. In this report, compounds containing a piperazine at ring C were synthesized in order to improve their water solubility through adding an alkaline group. Compounds with a benzoloxy group at ring C and a methylene linker at N were also prepared based on previous SAR results.

## 2. Results and Discussion

### 2.1. Synthesis of Permethyl Ningalin B Analogues

**Scheme 1:**
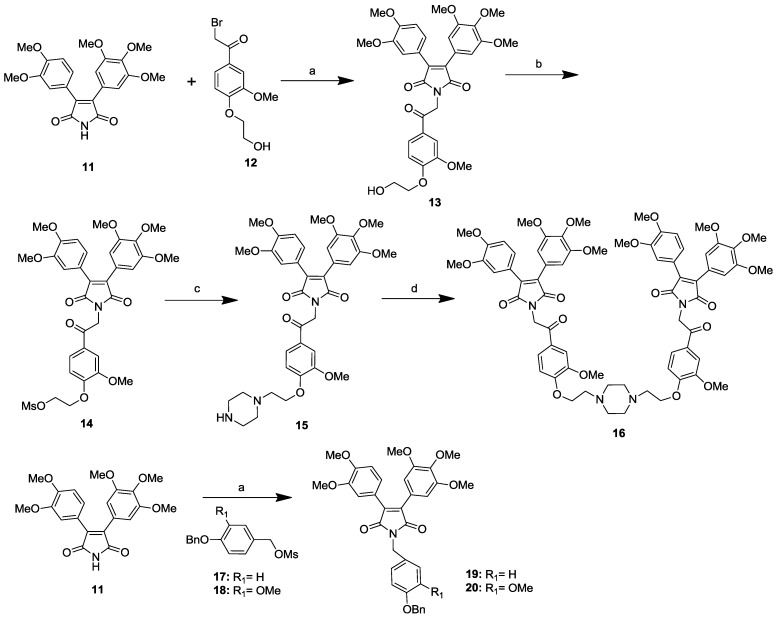
Synthetic route of compounds **15**, **16**, **19**, and **20**.

The permethyl ningalin B analogues containing a piperazine substituent were synthesized as shown in Scheme 1. Starting material **11**, which has been prepared and reported previously [23], was reacted with compound **12** in the presence of K_2_CO_3_ in DMF to afford intermediate **13**. Compound **13** was methanesulfonylated to provide methanesulfonylated intermediate **14**. Coupling of one equivalent **14** with ten equivalents piperazine produced the target molecule **15**. The target compound **16** was obtained from the reaction of **15** with one equivalent intermediate **14** or two equivalents **14** with one equivalent piperazine. 

Permethyl ningalin B analogues **19** and **20** possessing a benzoloxy group at ring C and a methylene linker at N were also synthesized and shown in Scheme 1. Starting material **11** was reacted with **17** or **18** in the presence of K_2_CO_3_ in DMF to give target compounds **19** and **20**, respectively.

Reagents and conditions: (a) K_2_CO_3_, DMF, rt, N_2_, overnight; (b) Et_3_N, methanesulfonyl chloride, CH_2_Cl_2_, 4 h; (c) K_2_CO_3_, piperazine, acetonitrile, reflux, 15 h; (d) K_2_CO_3_, DMF, 60 °C, overnight.

### 2.2. P-gp Modulating Activity of Permethyl Ningalin B Analogues

P-gp transfected breast cancer cell line (MDA435/LCC6MDR) and its parent (MDA435/LCC6), and human leukemia cell line K562/P-gp and its parent (K562) were employed. The LCC6MDR cells were about 90.4-fold more resistant to paclitaxel than its parental LCC6 cells (Table 1). K562/P-gp cells show about 279-fold higher resistance to paclitaxel than its wild type K562 cells (Table 1). A relatively low concentration of permethyl ningalin B analogues (1 μM) was used because of their high potency. There was no cytotoxicity towards cancer cells at such low concentration of permethyl ningalin B analogues (Table 1). Verapamil, the first-generation of P-gp modulator, displayed a moderate P-gp modulating activity with a RF of 3.8 in LCC6MDR cells (Table 1). On the contrary, PSC833, a potent P-gp modulator, showed very promising P-gp modulating activity with a RF of 80.3 in LCC6MDR cells and 520.9 in K562/P-gp cells.

In order to study their structure-activity relationship, twelve permethyl ningalin B analogues were divided into two series in Table 1. Compounds **3**–**7** and **8**–**10** have been reported previously [23,24]. In the present study, the new synthetic compounds **15** and **16** were further designed by addition of a piperazine group at acryl ring C. However, they exhibited no P-gp inhibition in both P-gp transfected cell lines when compared to the reported compounds **3**–**7** in series I. The bivalent flavonoid homodimers have been reported to exhibit potent P-gp and MRP-1 modulating activity [26,27,28,29]. Compound **16** was designed to possess bivalent-like structure. Nevertheless, analogue **16** did not show any P-gp modulating activity because of its shorter length of linker as compared to reported flavonoid dimers [26,27,28,29], and unfavorable position at ring C for joining two ningalin B molecules.

**Figure 2:**
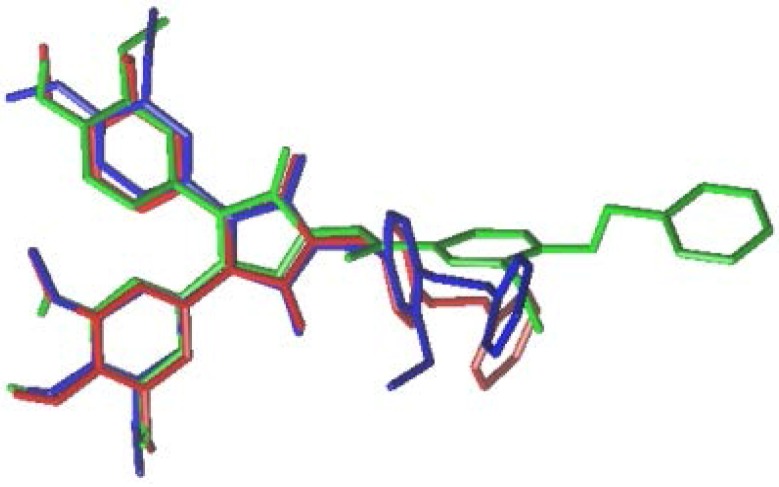
Alignments of compounds **7** (green) with **19** (red) and **20** (blue).

In series II, both compounds **19** (RF = 10.1 in LCC6MDR and RF = 136.3 in K562/P-gp) with a benzoloxy group at C-ring and **20** (RF = 13.1 in LCC6MDR cells and RF = 72.3 in K562/P-gp) with a benzoloxy and a methoxy group at C-ring exhibited moderate P-gp inhibition. They exhibited about 2.7 and 3.4-fold stronger potency than verapamil. However, they showed about 3.8- to 8.0-fold weaker P-gp modulating activity as compared to PSC833 (Table 1). They displayed similar P-gp modulating activity as the reported compounds **8**–**10** (RF = 9.3–18.2) with same methylene linker at N. However, when comparing to compound **7** which contains carbonylmethylene linker at N, they showed weaker P-gp modulating activity. After energy optimization and alignment, it was shown that molecules **19** (red) and **20** (blue) are shorter than **7** (green), and they possibly do not bind to P-gp as specifically as the compound **7** (Figure 2). This result suggests that polarity of linker at N plays an important role in determining P-gp modulating activity.

**Table 1:** P-gp modulating activity and cytotoxicity of permethyl ningalin B analogues.

Series	Cpds (1 μM)	No. of Methoxy Group on Acryl A Ring	No. of Methoxy Group on Acryl B Ring	Substituents at Acryl Ring C	Type of Linker	Cytotoxicity (IC_50_, μM)									LCC6MDR				K562/P-gp			
						LCC6			LCC6MDR			L929			Mean IC_50_ of Paclitaxel (nM)			RF	Mean IC_50_ of Paclitaxel (nM)			RF
	**3**	di	di	mono-methoxy	carbonylmethylene	/			/			/						9.9 ^a,b^	/			/
	**4**	di	di	di-methoxy	carbonylmethylene	/			/			/						8.2 ^a,b^	/			/
	**5**	di	di	tri-methoxy	carbonylmethylene	/			/			/						9.3 ^a,b^	/			/
**Ι**	**6**	di	di	mono-methoxy and mono-benzoloxy	carbonylmethylene	>100 ^b^			>100 ^b^			>100 ^b^						42.7 ^b^	/			/
	**7**	di	tri	mono-methoxy and mono-benzoloxy	carbonylmethylene	>100 ^b^			>100 ^b^			>100 ^b^						42.7 ^b^	/			/
	**15**	di	tri	piperzine	carbonylmethylene	31.2	±	2.1	30.7	±	0.6	44.6	±	4.7	209.6	±	22.1	0.7	539.1	±	76.7	1.1
	**16**	di	tri	piperzine	carbonylmethylene	>100			>100			>100			137.8	±	7.7	1.0	458.4	±	30.4	1.3
	**8**	di	di	mono-methoxy	methylene	/			/			/						9.3 ^a,b^	/			/
	**9**	di	di	di-methoxy	methylene	/			/			/						12.4 ^a,b^	/			/
**ΙΙ**	**10**	di	di	tri-methoxy	methylene	/			/			/						18.2 ^a,b^	/			/
	**19**	di	tri	mono-benzoloxy	methylene	>100			>100			>100			14.30	±	2.3	10.1	4.3	±	1.0	136.3
	**20**	di	tri	mono-methoxy and mono-benzoloxy	methylene	>100			>100			>100			11.00	±	1.7	13.1	8.1	±	2.0	72.3
/	Verapamil	/	/	/	/	63.9	±	1.7	63.8	±	0.1	89.2	±	8.2	38.0	±	7.0	3.8	/			/
/	PSC833	/	/	/	/	14.6	±	2.2	25.3	±	4.3	>100			1.8	±	0.3	80.3	1.1	±	0.5	520.9
/	LCC6MDR ^c^	/	/	/	/										144.6	±	9.4	1.0	/			/
/	LCC6 ^c^	/	/	/	/										1.6	±	0.3	90.4	/			/
/	K562/P-gp	/	/	/	/										/			/	586.0	±	123.8	1.0
/	K562	/	/	/	/										/			/	2.1	±	0.0	279.0

The relative fold (RF) represents the fold change in paclitaxel sensitivity of LCC6MDR cells in the presence of modulators relative to cells without modulators. RF = (IC_50_ without modulator)/(IC_50_ with modulator). The IC_50_ value from LCC6MDR containing no modulators was used for normalization (RF = 1.0). Known P-glycoprotein modulators, verapamil and PSC833, are included for comparison. Cytotoxicity of modulators alone towards LCC6, LCC6MDR and L929 (mouse fibroblast) were also determined. *N* = 2–3 independent experiments, and values are presented as the mean ± standard error of the mean. ^a,b^ These RF values and cytotoxicity values have been published [23,24]. ^c^ No modulator was used in LCC6MDR, LCC6, K562/P-gp and K562 cells. / = not determined.

### 2.3. Effect of Permethyl Ningalin B Analogues on Intracellular Rhodamine 123 and DOX Accumulation

**Figure 3:**
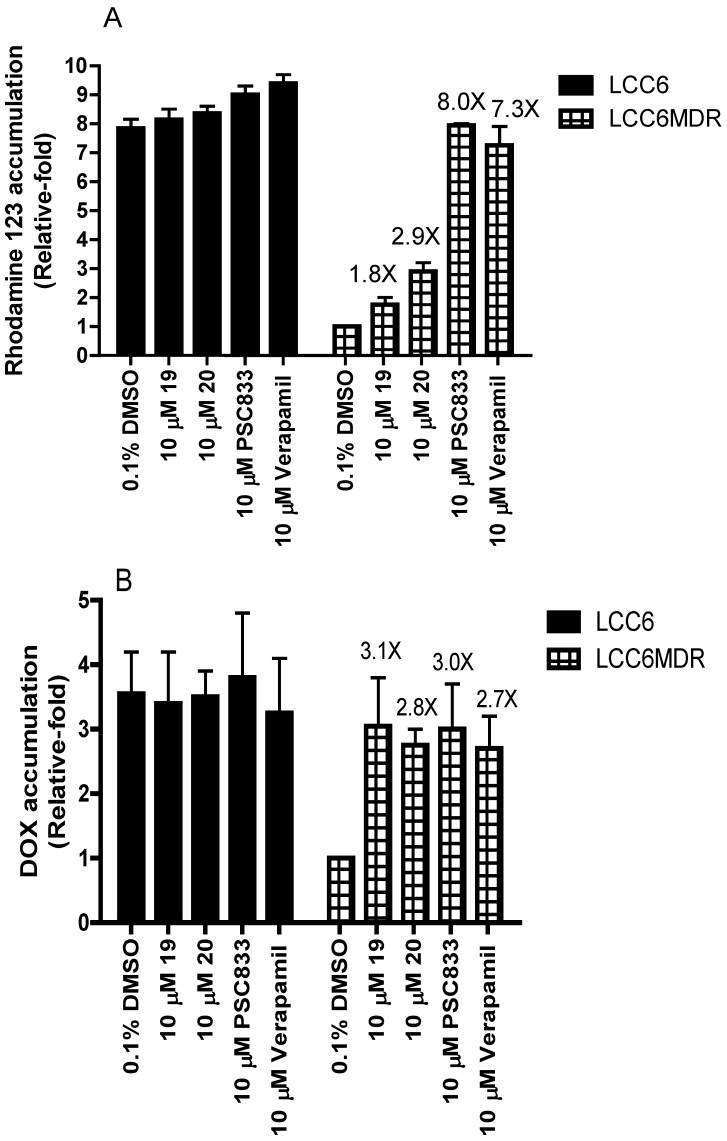
Effect of permethyl ningalin B analogues on rhodamine 123 (**A**) and DOX (**B**) accumulation in both LCC6 and LCC6MDR cells.

The above results showed that the compounds **19** and **20** are promising P-gp modulators. We are interested in investigating whether the modulation of P-gp-mediated drug resistance is associated with a concomitant increase in drug accumulation. Rhodamine 123 and DOX are known P-gp substrates and their fluorescent properties can help us to monitor intracellular drug accumulation. Here, PSC833 and verapamil were used as a positive control. The level of rhodamine 123 and DOX accumulation in LCC6 cells was about 7.8-fold and 3.6-fold higher than that of LCC6MDR, respectively (Figure 3A,B). This is because P-gp can pump rhodamine 123 and DOX out of the cells thereby lowering intracellular drug accumulation level. Treatment of LCC6MDR cells with 10 μM of PSC833 (8.0-fold) and verapamil (7.3-fold) can completely restore the rhodamine 123 accumulation level as its parent, whereas treatment with **19** (1.8-fold) and **20** (2.9-fold) can only result in a slight increase in rhodamine 123 accumulation (Figure 3A), suggesting that compounds **19** and **20** are weaker than PSC833 and verapamil in inhibiting P-gp pumping activity of rhodamine 123. In contrast, 10 μM of compounds **19** (3.1-fold) and **20** (2.8-fold) showed potent ability as PSC833 (3.0-fold) and verapamil (2.7-fold) in restoration of intracellular DOX accumulation in LCC6MDR cells (Figure 3B), suggesting that compounds **19** and **20** preferentially inhibit P-gp mediated DOX efflux rather than rhodamine 123 efflux. Overall, the mechanism of compounds **19** and **20** in reversing P-gp mediated drug resistance is by virtue of increasing the intracellular drug accumulation and eventually chemosensitizing LCC6MDR cells to anticancer drug again.

LCC6 or LCC6MDR cells were incubated with 10 μg/mL rhodamine 123 (**A**) or 20 μM DOX (**B**) at 37 °C for 2.5 h in the presence or absence of 10 μM of **19**, **20**, PSC833 and verapamil. 0.1% of DMSO was used as a negative control. After the incubation period, we lysed the cells and used the supernatant to measure the DOX or rhodamine 123 levels by spectrofluorometry. All fluorescence levels are normalized to 0.1% DMSO treated LCC6MDR cells. The values were presented as relative fold ± standard error of mean. Experiments were performed in duplicate and repeated twice.

## 3. Experimental Section

### 3.1. Materials and Methods

All moisture sensitive reactions were conducted under a nitrogen atmosphere in anhydrous, freshly distilled solvents. Starting materials and reagents, unless otherwise stated, were of commercial grades and were used without further purification. Literature procedures were used for the preparation of 3-(3,4-dimethoxyphenyl)-4-(3,4,5-trimethoxyphenyl)-1H-pyrrole-2,5-dione (**11**) [23], 4-(benzyloxy)benzyl methanesulfonate (**17**), 4-(benzyloxy)-3-methoxybenzyl methanesulfonate (**18**) [23,24]. All reactions were monitored by thin-layer chromatography (TLC), on aluminium sheets (Silica gel 60-F254, E. Merck, Darmstadt, Germany). Compounds were visualized by UV light. Column chromatography was carried out using silica gel (200–300 mesh). Melting points were recorded on a micro melting point apparatus MP-500D and were uncorrected. NMR spectra were recorded on a Jeol JNM-ECP spectrometer at 500 MHz for ^1^H NMR and 126 MHz for ^13^C NMR with TMS as the internal standard. Chemical shifts are expressed in δ (ppm) and coupling constants (*J*) in Hz. High-resolution (ESI) MS spectra were performed with a QTOF-2 Micromass spectrometer.

### 3.2. Synthesis of Compounds **13**–**20**

3-(3,4-dimethoxyphenyl)-1-(2-(4-(2-hydroxyethoxy)-3-methoxyphenyl)-2-oxoethyl)-4-(3,4,5-trimethoxyphenyl)-1*H*-pyrrole-2,5-dione (**13**). A mixture of compound 11 (400 mg, 1.0 mmol), 2-bromo-1-(4-(2-hydroxyethoxy)-3-methoxyphenyl)ethanone 12 (350 mg, 1.2 mmol) and K_2_CO_3_ (700 mg, 5.0 mmol) in anhydrous DMF (30 mL) was stirred at room temperature under an N_2_ atmosphere for overnight. The resulting solution was poured into water (90 mL), extracted with CH_2_Cl_2_, washed with brine, dried over anhydrous MgSO_4_, and the solvent was removed under reduced pressure. The residue was purified by flash chromatography on silica gel (EtOAc/PE = 4/1, v/v) to afford the desired compounds 13 (480 mg, 76.8% yield) as red solid. mp 171–173 °C; ESI-MS; *m/z* [M + H]^+^ 608.2; ^1^H NMR (CDCl_3_, 500 MHz) δ: 7.61 (d, *J* = 8.2 Hz, 1 H), 7.50 (s, 1 H), 7.27 (s, 1 H), 7.09 (s, 1 H), 6.93 (d, *J* = 8.4 Hz, 1 H), 6.85 (d, *J* = 8.4 Hz, 1 H), 6.80 (s, 2 H), 5.04 (s, 2 H), 4.18 (m, 2 H), 4.01 (s, 2 H), 3.88 (d, *J* = 12.5 Hz, 9 H), 3.73 (s, 9 H); ^13^C NMR (CDCl_3_, 126 MHz) δ: 190.0, 170.7, 153.2, 150.5, 149.5, 148.6, 139.3, 135.6, 134.4, 127.9, 124.2, 123.9, 122.7, 121.1, 112.7, 111.9, 110.9, 110.5, 107.1, 77.3, 77.1, 76.8, 70.6, 61.0, 56.1, 55.8, 44.2.

2-(4-(2-(3-(3,4-dimethoxyphenyl)-2,5-dioxo-4-(3,4,5-trimethoxyphenyl)-2,5-dihydro-1*H*-pyrrol-1-yl)acetyl)-2-methoxyphenoxy)ethyl methanesulfonate (**14**). A mixture of compound 13 (400 mg, 0.67 mmol), Et_3_N (340 mg, 3.35 mmol) in dry CH_2_Cl_2_ (30 mL) was cooled to 0 °C with stirring for 30 min. Then to the resulting solution, was added methanesulfonyl chloride (230 mg, 2.0 mmol) and stirred for 4 h while the reaction was allowed to warm to room temperature naturally. The solution was diluted with CH_2_Cl_2_ (30 mL), washed with brine and dried over anhydrous MgSO_4_. The solvent was evaporated to dryness to give the crude residue. The residue was purified by flash chromatography on silica gel (EtOAc/PE = 4/1, v/v) to afford the desired compounds 14 (320 mg, 0.47 mmol) as red solid. Yield 70.2%; mp 130–132 °C; ESI-MS; *m/z* [M + H]^+^ 676.2; ^1^H NMR (CDCl_3_, 500 MHz) δ: 7.60 (dd, *J* = 1.6, 8.4 Hz, 1 H), 7.51 (d, *J* = 1.7 Hz, 1 H), 7.27 (m, 1 H), 7.07 (d, *J* = 1.7 Hz, 1 H), 6.90 (d, *J* = 8.4 Hz, 1 H), 6.85 (d, *J* = 8.4 Hz, 1 H), 6.79 (s, 2 H), 5.03 (s, 2 H), 4.62 (m, 2 H), 4.32 (m, 2 H), 3.86 (m, 9 H), 3.69 (d, *J* = 3.0 Hz, 9 H), 3.13 (s, 3 H); ^13^C NMR (CDCl_3_, 126 MHz) δ: 190.0, 170.6, 153.1, 152.4, 150.6, 149.6, 148.6, 139.3, 135.6, 134.4, 128.4, 124.1, 123.9, 122.5, 121.1, 112.7, 111.9, 110.8, 107.1, 77.4, 77.1, 76.8, 67.9, 66.7, 60.9, 56.1, 55.9, 51.1, 44.2, 37.7.

3-(3,4-dimethoxyphenyl)-1-(2-(3-methoxy-4-(2-(piperazin-1-yl)ethoxy)phenyl)-2-oxoethyl)-4-(3,4,5-trimethoxyphenyl)-1*H*-pyrrole-2,5-dione (**15**). A mixture of compound 14 (200 mg, 0.3 mmol), piperazine (260 mg, 3.0 mmol) and K_2_CO_3_ (420 mg, 3.0 mmol) in anhydrous acetonitrile. After the addition, it was heated at reflux for about 15 h. The completion of the reaction was monitored by TLC. The resulting solution was poured into water (30 mL), extracted with CH_2_Cl_2_, washed with brine, dried over anhydrous MgSO_4_, and the solvent was removed under reduced pressure. The residue was purified by flash chromatography on silica gel (CH_2_Cl_2_/CH_3_OH/Et_3_N = 10/5/1, v/v) to afford the desired compounds **15** (100 mg, 0.3 mmol) as red solid. Yield 47.8%; mp 170–172 °C; ^1^H NMR (CDCl_3_, 500 MHz) δ: 7.62 (dd, *J* = 1.8, 8.3 Hz, 1 H), 7.52 (d, *J* = 1.8 Hz, 1 H), 7.27 (dd, *J* = 2.3, 8.9 Hz, 1 H), 7.10 (d,* J* = 1.8, 1 H), 6.94 (d, *J* = 8.4 Hz, 1 H), 6.86 (d, *J* = 8.4 Hz, 1 H), 6.81 (s, 2 H), 5.04 (s, 2 H), 4.23 (t, *J* = 6.1, 2 H), 3.89 (m, 9 H), 3.73 (s, 9 H), 2.90 (m, 8 H), 2.61 (s, 4 H); ^13^C NMR (CDCl_3_, 126 MHz) δ:190.0, 170.6, 153.4, 153.15, 150.6, 149.6, 148.6, 135.6, 134.5, 124.2, 123.9, 122.7, 121.1, 112.7, 111.5, 110.9, 110.6, 107.2, 77.3, 77.0, 76.8, 63.4, 60.9, 57.2, 55.9, 54.5, 45.9, 45.7, 44.2; HRMS calcd for (C_36_H_41_O_10_N_3_ + H)^+^ 676.2862, found 676.2855.

1,1′-((((piperazine-1,4-diylbis(ethane-2,1-diyl))bis(oxy))bis(3-methoxy-4,1-phenylene))bis(2-oxoethane-2,1-diyl))bis(3-(3,4-dimethoxyphenyl)-4-(3,4,5-trimethoxyphenyl)-1*H*-pyrrole-2,5-dione) (**16**). A suspension of compound 14 (0.12 g, 0.18 mmol), 15 (100 mg, 0.15 mmol), K_2_CO_3_ (100 mg, 0.75 mmol) was stirred in anhydrous DMF (10 mL) and heated to 60 °C for overnight. The solution was poured into ice-water (100 mL), and the resulting mixture was extracted with CH_2_Cl_2_. The organic layer was dried over anhydrous MgSO_4_. The solvent was evaporated to dryness, and the resulting residue was purified by flash chromatography using silica gel as the stationary phase and using CH_2_Cl_2_/CH_3_OH/Et_3_N (20:5:1) as the mobile phase to provide the compounds **16** (50 mg, 0.04 mmol) as a red solid. Yield 27.8%; mp 124–126 °C; ^1^H NMR (CDCl_3_, 500 MHz) δ: 7.62 (dd, *J* = 1.9, 8.4 Hz, 2 H), 7.53 (d,* J* = 1.9 Hz, 2 H) 7.28 (dd, *J* = 1.9, 8.4 Hz, 2 H) 7.11 (d,* J* = 1.9 Hz, 2 H) 6.95 (d, *J* = 8.5 Hz, 2 H) 6.87 (d, *J* = 8.5 Hz, 2 H) 6.82 (s, 4 H) 5.05 (s, 4 H) 4.26 (s, 4 H) 3.90 (d, *J* = 2.5 Hz, 12 H) 3.88 (s, 6 H) 3.72 (s, 18 H) 2.94 (s, 4 H) 2.72 (s, 8 H); ^13^C NMR (CDCl_3_, 126 MHz) δ: 190.0, 170.7, 153.2, 150.6, 149.5, 148.6, 124.2, 123.9, 122.6, 121.1, 112.7, 111.0, 110.9, 110.5, 107.2, 77.3, 77.0, 70.8, 61.0, 56.6, 55.9, 45.8, 44.9, 44.2; HRMS calcd for (C_68_H_72_O_20_N_4_ + H)^+^ 1265.4789, found 1265.4800.

### 3.3. General Procedure for the Preparation of the Compounds **19**, **20**

Under an N_2_ atmosphere, to a solution of compound **11** (100 mg, 0.25 mmol) and K_2_CO_3_ (138 mg, 1.00 mmol) in dry DMF (10 mL), 1.2 equivalent of the corresponding methanesulfonate derivates was added at room temperature. The solution was stirred for overnight. The solution was poured into ice-water (100 mL), and the resulting mixture was extracted with CH_2_Cl_2_, The organic layer was dried over anhydrous MgSO_4_. The solvent was evaporated to dryness, and the resulting residue was purified by flash chromatography to afford the titled compounds **19**, **20**. The physical and spectral data for **19**, **20** are listed below.

**1-(2-(4-(benzyloxy)phenyl)-2-oxoethyl)-3-(3,4-dimethoxyphenyl)-4-(3,4,5-trimethoxyphenyl)-1*H*-pyrrole-2,5-dione (19).** Yield 52.3%; mp 142–144 °C; ^1^H NMR (CDCl_3_, 500 MHz) δ: 7.40 (m, 4 H), 7.36 (t, *J* = 7.4 Hz, 2 H), 7.30 (t,* J* = 7.1 Hz, 1 H), 7.25 (d, *J* = 8.6 Hz, 1 H), 7.07 (s, 1 H) 6.94 (d, *J* = 8.5 Hz, 2 H) 6.85 (d, *J* = 8.5 Hz, 1 H) 6.79 (s, 2 H) 5.02 (s, 2 H) 4.73 (s, 2 H) 3.87 (t, *J* = 6.3 Hz, 6 H) 3.71 (s, 9 H); ^13^C NMR (CDCl_3_, 126 MHz) δ: 170.6, 158.4, 153.1, 150.5, 148.6, 139.3, 135.9, 135.1, 133.9, 130.2, 129.0, 128.6, 127.9, 127.4, 124.2, 123.8, 121.2, 114.9, 112.7, 110.9, 107.2, 77.5, 77.3, 77.0, 69.9, 60.9, 56.1, 55.8, 41.3; HRMS calcd for (C_35_H_34_O_8_N + H)^+^ 596.2279, found 596.2275.

**1-(4-(benzyloxy)-3-methoxybenzyl)-3-(3,4-dimethoxyphenyl)-4-(3,4,5-trimethoxyphenyl)-1*H*-pyrrole-2,5-dione (20).** Yield 52.3%; mp 110–112 °C; ^1^H NMR (CDCl_3_, 500 MHz) δ: 7.41 (d, *J* = 7.2 Hz, 2 H), 7.34 (t, *J* = 7.4 Hz, 2 H) 7.28 (d, *J* = 7.4 Hz, 1 H), 7.24 (dd, *J* = 1.8, 8.4 Hz, 1 H), 7.04 (m, 2 H), 6.96 (dd, *J* = 1.6, 8.2 Hz, 1 H) 6.83 (dd, *J* = 8.6, 9.9 Hz, 2 H) 6.76 (s, 2 H) 5.12 (s, 2 H) 4.70 (s, 2 H) 3.88 (m, 9 H) 3.70 (d, *J* = 2.1 Hz, 9 H); ^13^C NMR (CDCl_3_, 126 MHz) δ: 170.6, 153.1, 150.5, 149.5, 148.6, 147.8, 139.3, 137.1, 135.2, 133.9, 129.6, 128.5, 127.8, 127.2, 124.2, 123.8, 121.3, 121.1, 119.2, 113.8, 112.7, 110.9, 107.1, 77.5, 77.2, 76.9, 70.9, 65.0, 60.9, 56.1, 55.8, 41.7; HRMS calcd for (C_36_H_36_O_9_N + H)^+^ 626.2385, found 626.2381.

### 3.4. Materials for Biological Studies

Verapamil, paclitaxel, DOX, PSC833 and rhodamine 123 were purchased from Sigma-Aldrich. Dulbecco’s modified Eagle’s medium (DMEM), trypsin-ethylenediaminetetracetic acid (EDTA), and penicillin/streptomycin were from Gibco BRL. Fetal bovine serum (FBS) was from Hyclone Laboratories. 2-(4,5-Dimethylthiazol-2-yl-)-5-[3-(carboxymethoxy) phenyl]-2-(4-sulfophenyl)-2*H*-tetrazolium (MTS) and phenazine methosulfate (PMS) were purchased from Promega. Human breast cancer cell lines MDA435/LCC6 and MDA435/LCC6MDR were kindly provided by Dr. Robert Clarke (Georgetown University, Washington, DC). K562/P-gp and K562 human leukemia cell lines were a generous gift from Dr. Kenneth To (The Chinese University of Hong Kong, Hong Kong). L929 (mouse fibroblast) was from ATCC.

### 3.5. Cell Culture

MDA435/LCC6, MDA435/LCC6MDR, K562/P-gp, K562 and L929 cells were cultured in supplemented DMEM media containing 10% heat inactivated FBS and 100 U/mL penicillin and 100 μg/mL of streptomycin at 37 °C with 5% CO_2_ as described previously [26].

### 3.6. Cell Proliferation Assay

Six thousand cells of LCC6 or LCC6MDR and paclitaxel (0, 1.6, 4.9, 14.8, 44.4, 133.4, 400 nM) were mixed with or without modulators to a final volume of 200 μL in each well of 96-well plates as described previously [26]. After 5-day incubation, MTS (2 mg/mL) and PMS (0.92 mg/mL) were mixed in a ratio of 20:1. An aliquot (10 μL) of the freshly prepared MTS/PMS mixture was added into each well, and the plate was incubated for 2 h at 37 °C. Optical absorbance at 490 nm was then recorded with microplate absorbance reader (Bio-Rad, Hercules, CA, USA). IC_50_ values were calculated from the dose-response curves of MTS assays (Prism 4.0) [26]. 10,000 cells of K562 or K562/P-gp and paclitaxel (0, 0.64, 3.2, 16, 80, 400 and 2000 nM) were mixed with or without modulators to a final volume 100 μL in each well of 96-well plates and incubated for 3 days. After incubation, the % of survivors was determined using a mixture of MTS/PMS as mentioned previously.

### 3.7. Cytotoxicity Assay

Ten thousand cells of LCC6, LCC6MDR or L929 were incubated with various concentrations of modulators alone in a volume of 100 μL in each well of 96-well plate. After 3-day incubation, the % of survivors was determined using a mixture of MTS/PMS as mentioned previously. 

### 3.8. Rhodamine 123 and DOX Accumulation Assay

A total of 1 × 10^6^ cells of LCC6 and LCC6MDR were incubated at 37 °C for 2.5 h with 10 μg/mL rhodamine 123 or 20 μM DOX in the presence or absence of 10 μM modulators including compounds **19**, **20**, PSC8333 and verapamil. After incubation, the cells were washed with PBS and lysed with lysis buffer. The supernatant was saved and seeded in a black 96-well plate. The fluorescence level of DOX was determined by fluorescence spectrophotometer (BMG Technologies) using an excitation and an emission wavelength pair of 460 nm and 610 nm. The rhodamine 123 level was determined at wavelength of 480 nm for excitation and 520 nm for emission.

### 3.9. Calculated Molecular Descriptors

Calculated descriptors were determined with SYBYL-X 2.0. The structures of compounds **7**, **19** and **20** were built and energy minimized under the Tripos force field at 0.05 kcal/(mol Å). The Gasteiger-Huckel method was used to calculate the charges. Energy minimization was performed by the Powell method with 2000 iterations.

## 4. Conclusions

In this study, we have synthesized and evaluated P-gp modulating activity of two new compounds from series I containing a piperazine and two new compounds from series II with a mono-benzoloxy group at ring C. Compounds **15** with an additional piperazine at ring C did not show any P-gp inhibition, when compared to the reported compounds **3**–**7** with methoxys at ring C. The bivalent nature of compound **16** exhibited no P-gp modulating activity, implying that further modification is needed by adding a more basic functional group at ring C or synthesizing dimes at ring A or ring B. The P-gp modulating activity of compounds **19** and **20** with methylene linker at N are not as strong as the reported compound **7** with carbonylmethylene linker, indicating that polar linker is more favorable for making potent P-gp chemosensitizer. These two notoxic analogues of permethyl ningalin B may be potentially used as effective modulators of P-gp-mediated drug resistance in cancer cells.
